# *Candida metrosideri* pro tempore sp. nov. and *Candida ohialehuae* pro tempore sp. nov., two antifungal-resistant yeasts associated with *Metrosideros polymorpha* flowers in Hawaii

**DOI:** 10.1371/journal.pone.0240093

**Published:** 2020-10-08

**Authors:** Joon Klaps, Clara de Vega, Carlos M. Herrera, Robert R. Junker, Bart Lievens, Sergio Álvarez-Pérez

**Affiliations:** 1 Department of Microbial and Molecular Systems, Laboratory for Process Microbial Ecology and Bioinspirational Management, KU Leuven, Leuven, Belgium; 2 Department of Plant Biology and Ecology, Universidad de Sevilla, Sevilla, Spain; 3 Estación Biológica de Doñana, Consejo Superior de Investigaciones Científicas (CSIC), Sevilla, Spain; 4 Department of Biosciences, University Salzburg, Salzburg, Austria; 5 Evolutionary Ecology of Plants, Faculty of Biology, Philipps-University Marburg, Marburg, Germany; 6 Department of Animal Health, Faculty of Veterinary Medicine, Universidad Complutense de Madrid, Madrid, Spain; 7 Veterinary Clinical Hospital, Universidad Complutense de Madrid, Madrid, Spain; Leibniz Institut - Deutsche Sammlung von Mikroorganismen und Zellkulturen GmbH, GERMANY

## Abstract

Flowers produce an array of nutrient-rich exudates in which microbes can thrive, making them hotspots for microbial abundance and diversity. During a diversity study of yeasts inhabiting the flowers of *Metrosideros polymorpha* (Myrtaceae) in the Hawai’i Volcanoes National Park (HI, USA), five isolates were found to represent two novel species. Morphological and physiological characterization, and sequence analysis of the small subunit ribosomal RNA (rRNA) genes, the D1/D2 domains of the large subunit rRNA genes, the internal transcribed spacer (ITS) regions, and the genes encoding the largest and second largest subunits of the RNA polymerase II (*RPB1* and *RPB2*, respectively), classified both species in the family *Metschnikowiaceae*, and we propose the names *Candida metrosideri* pro tempore sp. nov. (JK22^T^ = CBS 16091 = MUCL 57821) and *Candida ohialehuae* pro tempore sp. nov. (JK58.2^T^ = CBS 16092 = MUCL 57822) for such new taxa. Both novel *Candida* species form a well-supported subclade in the *Metschnikowiaceae* containing species associated with insects, flowers, and a few species of clinical importance. The ascosporic state of the novel species was not observed. The two novel yeast species showed elevated minimum inhibitory concentrations to the antifungal drug amphotericin B (>4 μg/mL). The ecology and phylogenetic relationships of *C*. *metrosideri* and *C*. *ohialehuae* are also discussed.

## Introduction

Flowers have been increasingly recognized as unexplored reservoirs of yeast diversity due to the production of nutrient-rich exudates, such as floral nectar or stigmatic secretions, in which microbes can thrive [1–3]. Study of yeast diversity in flowers has led during the last decade to the discovery of more than 50 new species of ascomycetes yeasts, dispersed across different continents [2, 4–14]. Additionally, the taxonomic status of several flower-inhabiting yeast species still remains unclear because their phylogenetic affiliation is not yet satisfactorily resolved by available multilocus sequences, and/or they seem unable to produce sexual structures under standard laboratory conditions, making accurate classification challenging. This group of species with uncertain taxonomic placement includes several representatives from the family *Metschnikowiaceae* currently classified within the anamorphic genus *Candida* [11], such as *Candida auris* and the members of the *Candida haemulonis* species complex, which are closely related to the genus *Clavispora*. Notably, *C*. *auris* and some members of the *C*. *haemulonis* species complex are currently receiving increased attention because of their human pathogenic potential [15–17]. Additionally, the aforementioned species often show elevated minimum inhibitory concentrations (MICs) to multiple antifungals [15–21].

Recent mycological surveying of the flowers of the endemic Hawaiian plant species *Metrosideros polymorpha* (called *ʻōhiʻa lehua* in the vernacular language) [22] resulted in the cultivation of several isolates of uncertain identity. Sequence analysis of the D1/D2 regions of the large subunit (LSU) rRNA gene and the internal transcribed spacer (ITS) regions showed that the isolates represent two potential novel species from the family *Metschnikowiaceae* which are genetically distinct from any currently recognized species. The aim of this study was to characterize these novel yeast taxa, including their antifungal susceptibility profile, and to formally describe them as *Candida metrosideri* pro tempore sp. nov. (JK22^T^ = CBS 16091 = MUCL 57821) and *Candida ohialehuae* pro tempore sp. nov. (JK58.2^T^ = CBS 16092 = MUCL 57822). In addition, we discuss the phylogenetic relationships, antifungal susceptibility, and ecology of these new species.

## Materials and methods

### Isolates

The flower samples used in this study were collected in 2013 at Hawai’i Volcanoes National Park (Hawaii Island, HI, USA). These samples were part of a larger sample collection from a previous study that aimed to assess the bacterial diversity and community composition associated with the plant species *Metrosideros polymorpha* Gaud. (Myrtaceae) along an environmental and elevational gradient (see details in Junker and Keller [22]). *Metrosideros polymorpha* is found in all major islands of the Hawaiian archipelago, where it is distributed in a variety of habitats ranging from near the sea level to approx. 2500 m of altitude. Furthermore, *M*. *polymorpha* can grow on a broad range of substrates including recent lava flows and exhibits high resistance to different environmental stressors such as freezing, volcanic vapors, excessive moisture or dryness [23]. On the island of Hawaii, there are six varieties of *M*. *polymorpha* that differ in the morphology of vegetative and reproductive organs [24, 25], but our sampling focused exclusively on *M*. *polymorpha* var. *polymorpha*. Peak flowering occurs between February and July, although flowers can be found at any time of the year [26]. Floral visitors of this plant species are both native and introduced birds and insects, including bees, ants, and wasps [27–29], but these were not documented for the present study. As typical for bird-pollinated plants, the flowers of this species produce large amounts of nectar that is presented in cup-like receptacles. The average sugar concentration of *M*. *polymorpha* nectar is around 30% w/w [27].

A total of 91 floral samples were used in the present study, including nectar (*n* = 30), stamens (*n* = 31) and styles (*n* = 30) of 33 individual *M*. *polymorpha* trees from 14 populations. Five yeast isolates were characterized in this study: i) isolate JK22^T^, retrieved from the stamens of a flower collected in a first plant population (GPS coordinates: 19.67, -155.34; 1590 m above the sea level); and ii) isolates JK58.2^T^, JK58.4, JK58.5, and JK58.6, obtained from the styles of a single flower collected in a different population (19.37, -155.22; 999 m).

### Phylogenetic analyses

Genomic DNA was isolated from yeast colonies as described in Álvarez-Pérez *et al*. [30]. PCR amplification of the D1/D2 region of the LSU rRNA gene was done using the NL1 and NL4 primers [31], and sequences coding for the small-subunit (SSU) rRNA gene, ITS1, 5.8S rRNA gene and ITS2 were amplified with primers Fungi-18S-up and ITS4 [13]. In addition, the protein-encoding genes *RPB1* (largest subunit of RNA polymerase II) and *RPB2* (second largest subunit of RNA polymerase II) were amplified with primers RPB1-Af and RPB1-Cr and RBP2-5F and RPB2-7Cr, respectively [32]. In all cases, reaction mixtures contained 5 μL of Titanium Buffer (10X, Clontech), 0.25 mM MgCl_2_ (Thermo Scientific), 0.25 mM of each primer (Sigma-Aldrich), 0.5 U Titanium Taq polymerase (50X, Clontech), and 50 ng of template DNA in a final volume of 50 μL. All PCR amplifications were carried out in a BioRad T100 thermocycler and consisted of a denaturation step of 2 min at 95°C, followed by 35 cycles of 30 s at 94°C, 30 s at 52°C and 2 min at 72°C, and a final extension of 10 min at 72°C. PCR products were purified using Thermo Scientific’s GeneJET PCR Purification Kit and sent for two-way Sanger sequencing to Macrogen Europe (Amsterdam, The Netherlands). Sequencing primers were the same as those used for PCR amplification in all cases except for *RPB2*, for which the large amplicon length made necessary the use of four sequencing primers: RBP2-5F, RPB2-7Cr, RPB2-6F, and RPB2-6R [16]. Upon sequencing, DNA sequences were assembled and manually edited with the program SeqTrace [33]. Next, DNA sequences were aligned using Muscle [34] and the resulting alignments were used to build neighbor joining (NJ) and maximum likelihood (ML) phylogenetic trees, using MEGA X [35] and the online version of PhyML v3.0 (http://www.atgc-montpellier.fr/phyml/ [36]), respectively. NJ analyses were performed using Kimura’s two parameter model, whereas ML analyses were based on the best available model selected by jModeltest v2.1.10 [37]. In all cases, bootstrap support values were obtained from 1000 random resamplings. Consensus trees were visualized and edited in MEGA X.

### Phenotypic characterization

Yeast isolates were phenotypically characterized according to the standard procedures described by Kurtzman et al. [38]. Assays testing for fermentations and growth on nitrogen sources were performed in liquid media, and incubated at 25°C for up to 33 and 15 days, respectively. Assimilation of carbon compounds was assessed on agar plates by replica plating and incubated at 25°C for up to 7 days. Determination of the hemolytic activity of isolates was performed by streaking a single colony of each isolate on Columbia blood agar (VWR) and incubating the plates for 3 days at 25°C. The Gram-positive hemolytic bacterium *Staphylococcus aureus* ATCC 29213^T^ was used as a positive control in these tests, and the presence of a halo of blood digestion around the colonies was interpreted as a positive result. Growth under microaerobiosis was determined by culturing all isolates on potato dextrose agar (PDA, Neogen) and incubating the plates at 25°C for 5 days in a candle jar. Additionally, growth under anaerobic conditions was evaluated by streaking isolates on PDA and incubating the plates into a jar containing an anaerobiosis generator sachet and anaerobiosis indicator (Microbiology Anaerocult, Merck) for 4 days at 25°C. In both tests, the appearance of yeast colonies on the plates was recorded as a positive result. Further, colony morphology was determined using plate cultures grown on yeast malt agar (YM; 2.0% agar (Oxoid), 1.0% glucose (Sigma-Aldrich), 0.5% Bacto Peptone (Becton, Dickinson and Company, BD), 0.3% malt extract (Oxoid), 0.3% yeast extract (Oxoid), pH 6.2 ± 0.2) or PDA for 48–72 h at 25°C. Single colonies were examined with an SZX10 stereomicroscope (Olympus). Microscopic inspection of cells was performed with an optical microscope (DP74 Microscope Digital Camera, Olympus) using a phase contrast objective. Ascospore formation was analyzed on YM agar, 5% malt extract agar, diluted (1:9 and 1:19) V8 agar, restriction growth agar, Gorodkowa agar, and Custer’s chalk agar [38]. All these media were inoculated by spreading portions of yeast colonies on the agar surface using sterile plastic loops (VWR). Evaluation of mating compatibility was performed by mixing conspecific yeast isolates in every possible pair-wise combination [38], and inoculated plates were incubated for 7 weeks at 20 and 25°C. Since no conspecific isolates were available for JK22^T^, mating tests were not performed for this isolate.

Finally, given the close phylogenetic relatedness of the studied isolates with multi-drug resistant fungal pathogens such as *Candida auris* and *C*. *haemulonis* (see Results and discussion), we determined the *in vitro* antifungal susceptibility of such isolates by the commercially prepared Sensititre YeastOne YO10 colorimetric antifungal panel (Thermo Fisher). Antifungals included in this test were anidulafungin, amphotericin B, micafungin, 5-flucytosine, and a number of azoles including fluconazole, itraconazole, posaconazole and voriconazole. On the day of the assay, suspensions of 1.5–8×10^3^ cells/mL were prepared in YeastOne inoculum broth, and the dried panels were rehydrated by dispensing 100 μL of yeast suspension into each well. Assay plates were covered with adhesive seals and incubated at 25°C for 48 h. The MIC endpoints were defined as the lowest concentration of the antifungal drugs preventing the development of a red color (i.e. first blue or purple well). All isolates were tested twice on different days.

### Ethics

Ethical approval for collecting plant samples at Hawaii Volcanoes National Park was granted by the USA Department of the Interior -National Park Service on March 2014 (HAVO-2013-SCI-003). Accessed lands are protected.

### Nomenclature

The electronic version of this article in Portable Document Format (PDF) in a work with an ISSN or ISBN will represent a published work according to the International Code of Nomenclature for algae, fungi, and plants, and hence the new names contained in the electronic publication of a PLOS article are effectively published under that Code from the electronic edition alone, so there is no longer any need to provide printed copies.

In addition, new names contained in this work have been submitted to MycoBank from where they will be made available to the Global Names Index. The unique MycoBank number can be resolved and the associated information viewed through any standard web browser by appending the MycoBank number contained in this publication to the prefix http://www.mycobank.org/MB/. The online version of this work is archived and available from the following digital repositories: PubMed Central and Zenodo.

## Results and discussion

The overall frequency of yeasts occurrence in the samples analyzed (*n* = 91) was 55%. In total, 80 yeast isolates were obtained, out of which five isolates were representative of two undescribed species. Other yeasts retrieved from the flowers of *M*. *polymorpha* were identified as *Metschnikowia rancensis* (*n* = 21 isolates), *Aureobasidium melanogenum* (*n* = 15), *Starmerella bombicola* (*n* = 8), *Debaryomyces vindobonensis* (*n* = 7), *Rhizosphaera macrospora* (*n* = 5), *Wickerhamiella azyma* (*n* = 4), *Metschnikowia hawaiiana* (*n* = 2), *Papiliotrema terrestris* (*n* = 2), *Priceomyces melissophilus* (*n* = 2), *Curvibasidium pallidicorallinum* (*n* = 2), *Starmerella apicola* (*n* = 1), *Starmerella bombi* (*n* = 1), *Starmerella sorbosivorans* (*n* = 1), *Dimennazyma cisti-albidi* (*n* = 1), *Filobasidium globisporum* (*n* = 1), *Kodamaea ohmeri* (*n* = 1) and *Rhodotorula mucilaginosa* (*n* = 1).

Analysis of the D1/D2 region of the LSU rRNA gene (JK22^T^ = 522 bp, JK58.2^T^ = 515 bp) from the studied isolates revealed that all of them had a low sequence identity with the sequences included in GenBank (<93.5%). One of these isolates is described in the present article as *Candida metrosideri* sp. nov. (JK22^T^ = CBS 16091 = MUCL 57821), and the other four isolates as *Candida ohialehuae* sp. nov. (type strain: JK58.2^T^ = CBS 16092 = MUCL 57822) (Table 1). All isolates belonging to *C*. *ohialehuae* were retrieved from the styles of the same *M*. *polymorpha* flower, while the isolate representing *C*. *metrosideri* was found in the stamens of a different *M*. *polymorpha* flower. Furthermore, *C*. *metrosideri* was co-isolated with *Metschnikowia rancensis*, whereas *C*. *ohialehuae* was found to co-occur with *M*. *rancensis* and *Debaryomyces* *hansenii*.

**Table 1:** Isolation sources and GenBank accession numbers of the yeast isolates characterized in this study.

Species	Isolates	Isolation source^a^	GenBank accession numbers			
			LSU	ITS	*RPB1*	*RPB2*
*Candida metrosideri* sp. nov.	JK22^T^ = CBS 16091 = MUCL 57821	Stamen	MN240861	MN240806	MN239193	MN239194
*Candida ohialehuae* sp. nov.	JK58.2^T^ = CBS 16092 = MUCL 57822	Style	MN240860	MN240805	MN239192	MN239195
	JK58.4 = CBS 16093 = MUCL 57823	Style	MN240859	MN240804	MN239191	MN239196
	JK58.5 = CBS 16094 = MUCL 57824	Style	MN240858	MN240803	MN239190	MN239197
	JK58.6 = CBS 16095 = MUCL 57825	Style	MN240857	MN240802	MN239189	MN239198

^a^Floral part of *Metrosideros polymorpha* from which the isolate was obtained.

### Phylogenetic affiliation

*Candida ohialehuae* showed three D1/D2 intraspecific sequence variants. JK58.2^T^ differed in the D1/D2 sequence by one nucleotide substitution from isolates JK58.4, JK58.5, which have identical sequences, and isolate JK58.6 diverged by one gap. No polymorphism was detected among *C*. *ohialehuae* isolates for the ITS, *RPB1*, and *RPB2* sequences. Comparison of the D1/D2 region of the LSU rRNA gene obtained for *C*. *metrosideri* and *C*. *ohialehuae* revealed a total of 68 substitutions and 15 gaps (15.9% variation).

**Fig 1:**
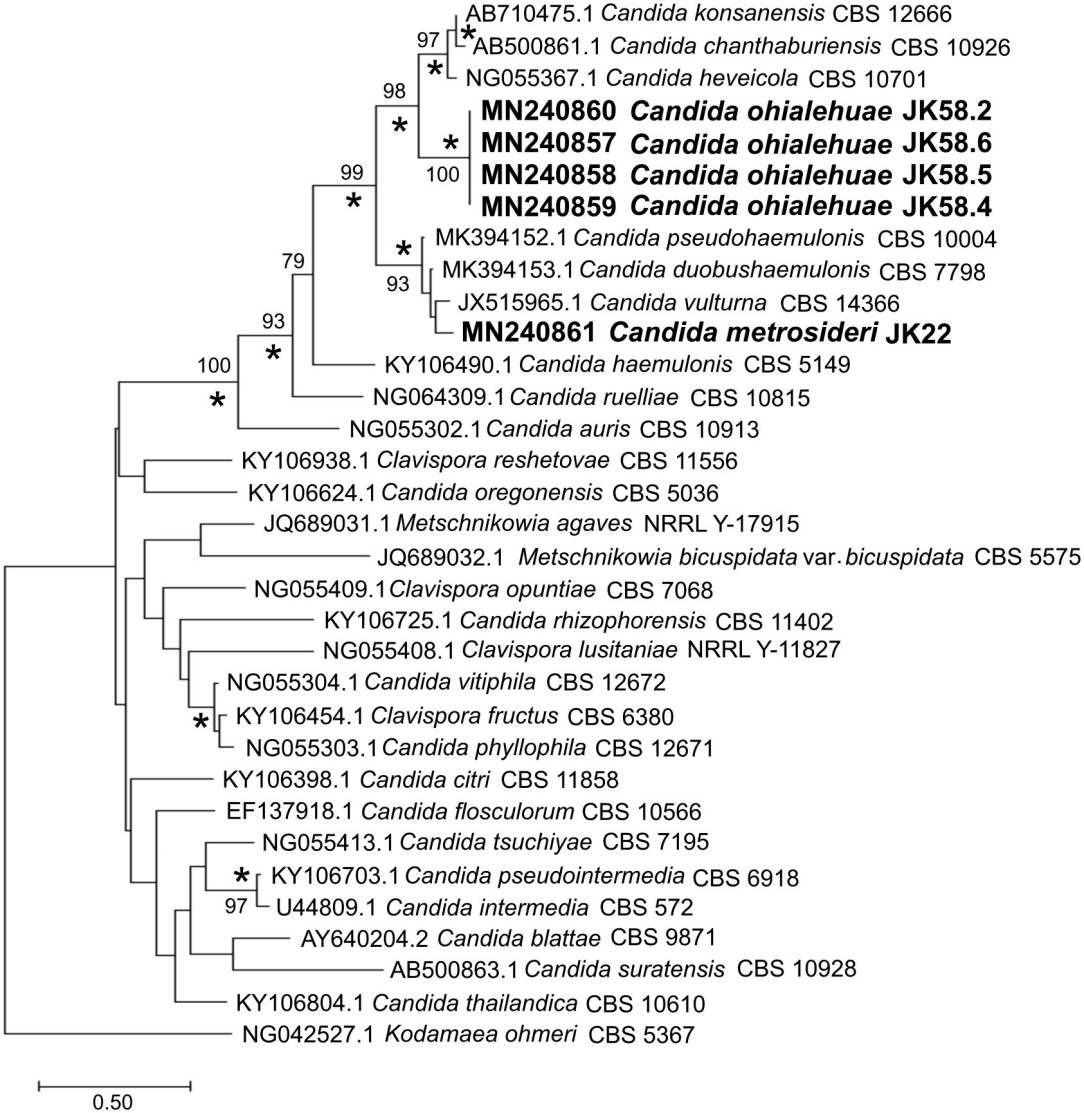
Phylogenetic placement of *Candida metrosideri* and *Candida ohialehuae* based on DNA sequence analysis of the D1/D2 domains of the large subunit (LSU) rRNA gene, as obtained by maximum likelihood analysis. Evolutionary distances were computed using the Hasegawa, Kishino and Yano (HKY85) model and are in the units of the number of base substitutions per site (see the scale). The rate variation among sites was modeled with a gamma distribution (shape parameter = 0.69) and a proportion of invariant sites (0.46). There was a total of 561 positions in the final alignment. Bootstrap node support values, based on 1000 replicates, ≥70% are shown next to the branches. Additionally, NJ (Kimura-2-parameter model, gamma distribution’s shape parameter = 0.69) bootstrap node support values ≥70% are marked by asterisks.

Phylogenetic analysis of the D1/D2 region of the LSU domain obtained by NJ and ML methods revealed that *C*. *metrosideri* clusters with *Candida duobushaemulonis*, *Candida vulturna*, and *Candida pseudohaemulonis*, whereas *C*. *ohialehuae* forms a well-supported sister clade with *Candida konsanensis* (42 substitutions and 10 indels, 9.7% variation), *Candida heveicola* (45 substitutions and 8 indels, 10.3% variation), and *Candida chantaburiensis* (46 substitutions and 10 indels, 10.8% variation) (Fig 1 and Table 2). The clade including both new species and the aforementioned close relatives was found to be well supported (88% and 98.6% bootstrap in the NJ and ML trees, respectively).

**Table 2:** Sequence divergence from *Candida metrosideri* JK22^T^ to closely related species based on nucleotide sequences obtained from selected loci^a^.

Close relatives	LSU			ITS			*RPB1*			*RPB2*		
	SNS	Indels	%	SNS	Indels	%	SNS	Indels	%	SNS	Indels	%
*Candida doubushaemulonii*	26	8	6.5%	21	5	9.8%	22	0	3.0%	43	0	4.0%
*Candida vulturna*	34	16	9.5%	22	6	10.4%	ND	ND	ND	ND	ND	ND
*Candida pseudohaemulonis*	37	19	10.7%	24	11	13.0%	26	0	4.0%	51	0	5.0%

^a^LSU, D1/D2 domains of the large subunit rRNA gene; ITS, internal transcribed spacer regions; *RPB1* and *RPB2*, genes encoding the largest and second largest subunits of the RNA polymerase II, respectively. SNS, single nucleotide substitutions; %, percentage of sequence divergence.

**Fig 2:**
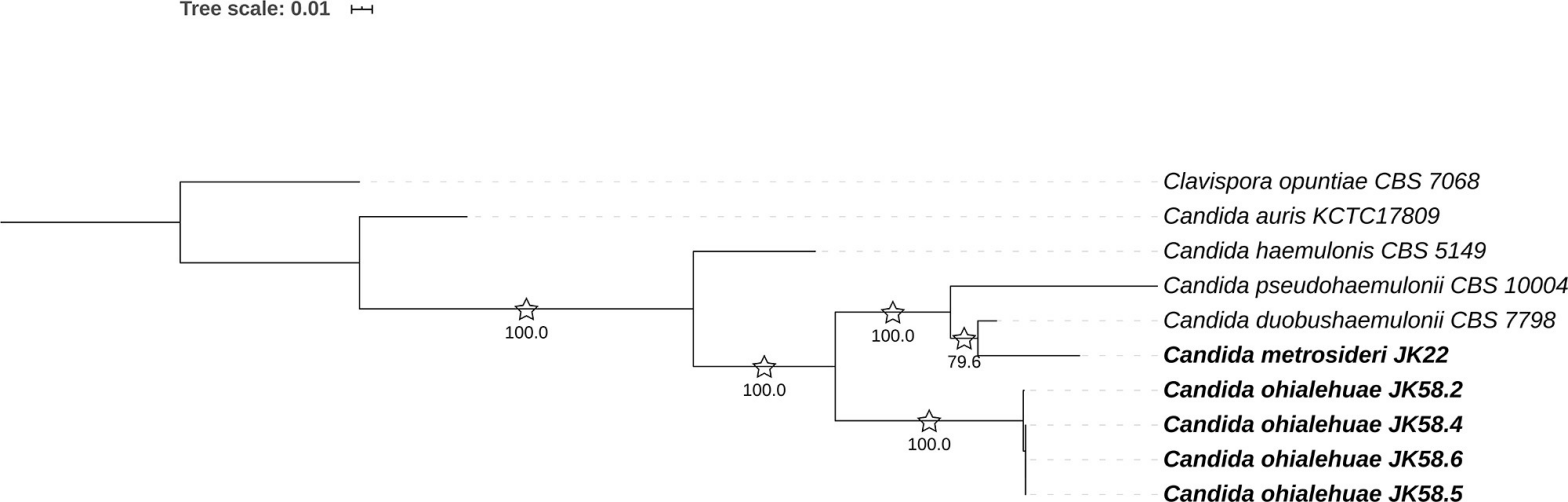
Phylogenetic placement of *Candida metrosideri* and *Candida ohialehuae* based on the analysis of concatenated LSU rRNA + ITS + *RPB1* + *RPB2* (2069 bp in total), as obtained by maximum likelihood. Evolutionary distances were computed using the general time-reversible (GTR) model and are in the units of the number of base substitutions per site (see the scale). The rate variation among sites was modeled with a gamma distribution (shape parameter = 0.39) and a proportion of invariant sites (0.14). Bootstrap node support values, based on 1000 replicates, ≥70% are shown next to the branches. Additionally, NJ (Kimura-2-parameter model, gamma distribution’s shape parameter = 0.28) bootstrap node support values ≥70% are marked by asterisks. The tree was rooted on *Clavispora opuntiae* CBS 7068.

Previous studies have shown that strains belonging to the same yeast species usually differ by less than 1% mismatches in the D1/D2 domain [31, 39, 40] (but see Lachance et al. [41] for a description of unusual intra-species polymorphism in this gene region in *Clavispora lusitaniae*). The significant sequence divergence in the D1/D2 region displayed by *C*. *ohialehuae* and *C*. *metrosideri* between them and with respect to previously described species suggests that these might deserve the consideration of new yeast taxa. Nevertheless, in order to confirm our taxonomic proposal and the phylogenetic placement of *C*. *metrosideri* and *C*. *ohialehuae*, we built additional phylogenies based on the ITS region (ITS1 + 5.8S + ITS2: JK22^T^, 242 bp; JK58.2^T^, 243 bp; S1 Fig), the *RPB1* (JK22^T^, 657 bp; JK58.2^T^, 657 bp; S2 Fig) and *RPB2* (JK22^T^, 1055 bp; JK58.2^T^, 1076 bp; S3 Fig) genes, and a concatenation of LSU rRNA + ITS + *RPB1* + *RPB2* sequences (Fig 2). Unfortunately, most clades observed in the ITS tree were only poorly supported (bootstrap values <70%; S1 Fig), and the absence of *RPB1* and *RPB2* sequences in public databases for several species closely related to the genus *Clavispora* hindered further confirmation of the phylogenetic placement of most taxa. Notably, the clustering of *C*. *metrosideri*, and *C*. *ohialehuae* with *C*. *doubushaemulonis*, *C*. *haemulonis* and *C*. *pseudohaemulonis* was confirmed by phylogenetic analysis of the LSU rRNA + ITS + *RPB1* + *RPB2* concatamer (Fig 2).

Although the phylogenetic analyses performed suggested that the new species described here belong to the family *Metschnikowiaceae* and are closely related to *Clavispora*, studied isolates cannot be unequivocally assigned to this genus. Additionally, previous studies have demonstrated that *C*. *auris* and *C*. *haemulonis* are distant from *Clavispora lusitaniae* and do not constitute a monophyletic group [20, 42]. Therefore, we propose to refer to these novel species as *Candida metrosideri* pro tempore sp. nov. and *Candida ohialehuae* pro tempore sp. nov. The term ‘pro tempore’ was proposed to indicate lineages that are temporary generic assignments in absence of further taxonomic evidence [20, 43].

### Phenotypic characterization

**Fig 3:**
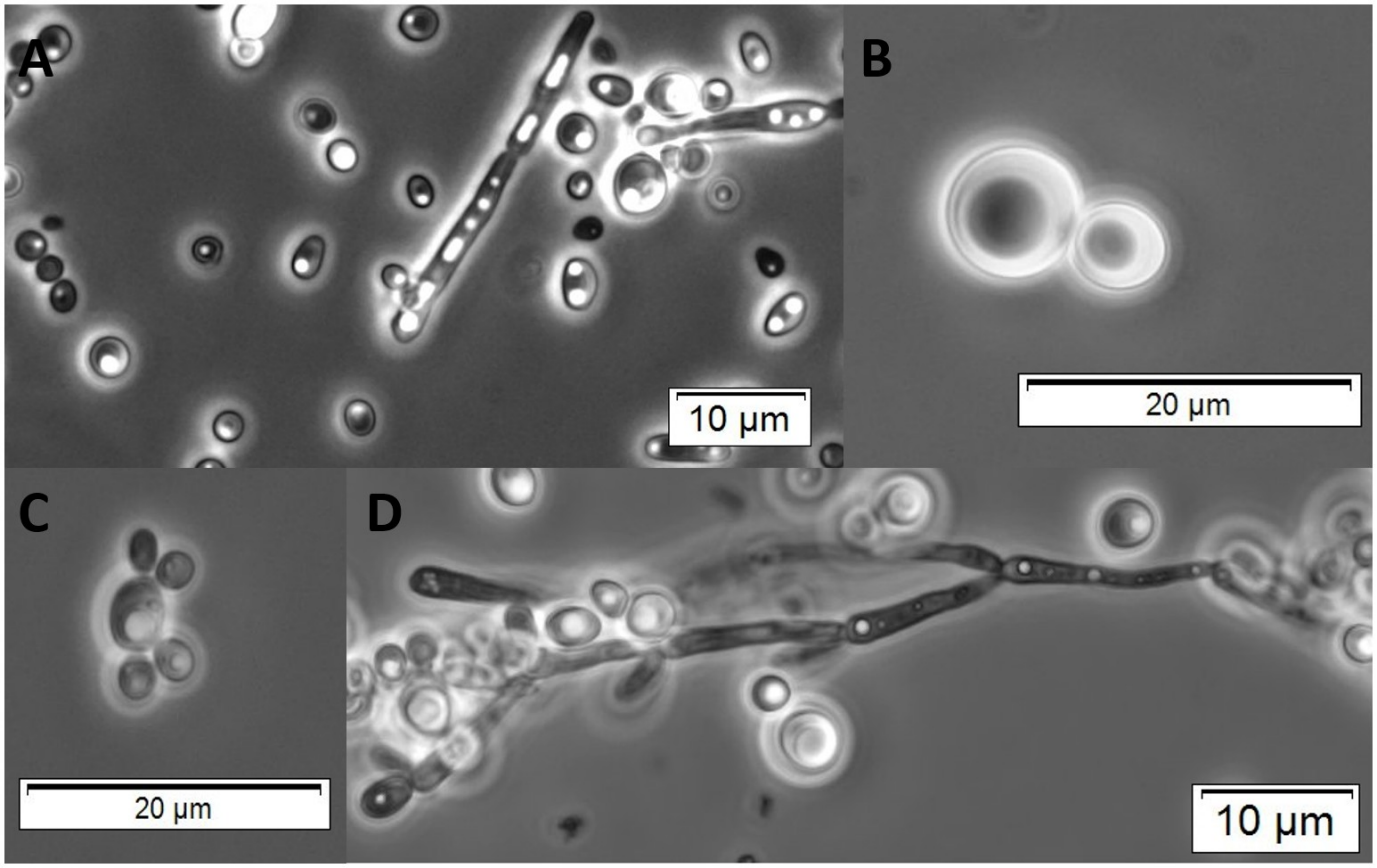
Micromorphology of *Candida metrosideri* and *Candida ohialehuae*. Multipolar budding and pseudohyphae of *Candida ohialehuae* JK58.2^T^ (A, C and D, respectively), and budding cells of *Candida metrosideri* JK22^T^ (B).

The results of the physiological tests for the novel species *C*. *metrosideri* and *C*. *ohialehuae*, and close phylogenetic relatives are shown in Table 3 and S1 Table, and the micromorphology of some of the studied isolates is shown in Fig 3. *Candida ohialehuae* ferments both glucose and sucrose as do most of its closest relatives, including *C*. *heveicola*, *C*. *konsanensis*, *C*. *vulturna*, and *C*. *duobushaemulonis*. In contrast, *C*. *metrosideri* and *C*. *chantaburiensis* can ferment glucose but not sucrose. Fermentation of fructose, which together with sucrose and glucose is a sugar commonly found in flowers (e.g. in floral nectar), was not observed for *C*. *metrosideri*, whereas *C*. *ohialehuae* showed a delayed positive reaction. Notably, *C*. *metrosideri* is unable to assimilate galactose, whereas the type strains of close phylogenetic relatives can assimilate this sugar. On the other hand, *C*. *ohialehuae* and *C*. *auris* cannot assimilate citric acid and succinic acid. Additionally, *C*. *ohialehuae* and *C*. *metrosideri* together with *C*. *vulturna* can assimilate potassium nitrate and sodium nitrite, whereas all other closely-related species cannot. Ethanol, which in floral microhabitats can be released by yeasts as a fermentation by-product [44], is not assimilated by *C*. *vulturna*, *C*. *ohialehuae* and *C*. *chantaburiensis*, but their closest relatives can. Both *C*. *metrosideri* and *C*. *ohialehuae* can endure strong osmotic pressures (up to 60% w/w for some tested isolatess) and high saline solutions (10% NaCl), traits which are shared by most of the closely related species. These traits allow the species to be well adapted to environments with low water activity. Finally, *C*. *metrosideri*, *C*. *ohialehuae* and their sister species *C*. *vulturna*, *C*. *heveicola* and *C*. *konsanensis* are able to grow in vitamin-free medium. Finally, ascospore production was not observed for any of the pure or mixed cultures of *C*. *ohialehuae* on common sporulation media (see Materials and methods) after eight weeks of incubation at 20 or 25°C.

**Table 3:** Phenotypic characteristics of *Candida metrosideri*, *Candida ohialehuae* and their close phylogenetic relatives. A complete version of this table is presented as S1 Table.

Characteristic	Reference strains						7	8
	1	2	3	4	5	6		
**Fermentation**								
Glucose	+	+	+	+	+	+	w	+
Sucrose	+	+	ND	+	+/w	-	-	d/s
**Assimilation**								
Fructose	ND	ND	ND	ND	ND	ND	+	+
Galactose	+	+	+	+	+	+	-	s
Methanol	-	w/d	-	-	-	-	-	-
Ethanol	-	w/d	d	+	d	-	+	-
**Growth on YM agar at**:								
30°C	+	+	ND	ND	+	+	+	+
37°C	+	+	+	+	+	w	-	-
**Other phenotypic tests**:								
Osmotolerance (50% glucose, w/w)	+	+	ND	ND	+	+	+	+
Osmotolerance (60% glucose, w/w)	+	+	ND	ND	w	+	-	w/s
Growth in vitamin-free medium	+	-	-	+	+	ND	s	s

Strains: 1, *Candida vulturna* CBS 14366^T^; *Candida doubushaemulonii* CBS 7798^T^; 3, *Candida pseudohaemulonii* CBS 10004^T^; 4, *Candida heveicola* CBS 10702^T^; 5, *Candida konsanensis* CBS 12666^T^; 6, *Candida chantaburiensis* CBS 10926^T^; 7, *Candida metrosideri* JK22^T^; 8, *Candida ohialehuae* JK58.2^T^. Data for the reference strains originates from references [16, 20, 21, 47, 48] and the CBS database (http://www.cbs.knaw.nl). All isolates were positive for assimilation of D-glucose, sucrose, trehalose, maltose, glycerol, D-mannitol, D-glucitol, and L-lysine, and negative for assimilation of lactose. All isolates grow at a temperature of 25°C. Scoring system: +, positive; -, negative; d, delayed positive (latent); s, slowly positive; w, weakly positive; v, variable; ND, no data available.

Thermotolerance and hemolytic activity are considered important virulence factors that help mammal pathogens to survive inside their hosts [45]. Tested isolates of *C*. *metrosideri* and *C*. *ohialehuae* did not display any hemolytic activity and were unable to grow at 37°C. In contrast, closely-related species such as *C*. *auris*, *C*. *ruelliae*, *C*. *haemulonis*, *C*. *duobushaemulonis*, *C*. *pseudohaemulonis*, *C*. *vulturna*, *C*. *heveicola*, *C*. *chantaburiensis*, and *C*. *konsanensis* can grow at 37°C, and most of them have been repeatedly isolated from clinical samples [16, 19–21, 46–49]. However, based on the study performed we cannot discard the possibility that *C*. *ohialehuae and C*. *metrosideri* might act as opportunistic pathogens of humans and/or animals that have niches outside clinical and endothermic environments in which they can thrive [50]. Such possibility should be tested in the future, e.g. by using vertebrate and/or invertebrate animal models of fungal infection [51, 52].

Due to the close phylogenetic relatedness of *C*. *metrosideri* and *C*. *ohialehuae* to other yeast species whose members often display resistance to multiple antifungals [16–21, 49], we analyzed the *in vitro* susceptibility of our isolates to amphotericin B, 5-fluorocytosine, and a number of azoles and echinocandins. The results of these tests (Table 4) revealed that *C*. *metrosideri* and *C*. *ohialehuae* are sensitive to azoles and 5-fluorocytosine. Anidulafungin and micafungin MICs were also relatively low for *C*. *metrosideri* (0.25 and 0.06 μg/mL, respectively), but moderately elevated for *C*. *ohialehuae* (1–2 and 0.5 μg/mL). In contrast, both species showed elevated MIC values for amphotericin B (≥4 μg/mL in all cases). Amphotericin B binds to ergosterol, inserts into the cytoplasmic membrane and forms pore-like structures resulting in osmotic instability, loss of membrane integrity, metabolic disruption, and ultimately cell death [53, 54]. Decreased susceptibility to amphotericin B is often caused by a decrease of the ergosterol content in the plasmalemma or a change in the target lipid as a consequence of mutations in the genes encoding the ergosterol biosynthesis pathway [55]. Nevertheless, resistance to amphotericin B often results in a decreased fitness and ability of strains to survive in animal hosts [56, 57].

**Table 4:** Antifungal susceptibility profile of the *Candida metrosideri* and *Candida ohialehuae* isolates characterized in this study.

	MIC (μg/mL)^a^							
Isolate	AFG	AMB	MFG	5-FC	POS	VRC	ITC	FLC
JK22^T^	0.25	4	0.06	<0.06	0.03	0.03	0.06	2
JK58.2^T^	1	>8	0.5	0.12	0.03	0.06	0.06	4
JK58.4	2	>8	0.5	≤0.06	0.015	0.015	0.06	2
JK58.5	1	4	0.5	0.06	0.015	0.015	0.03	2
JK58.6	1	>8	0.5	≤0.06	0.015	0.015	0.03	2

^a^Minimum inhibitory concentration (MIC) to: AFG, anidulafungin; AMB, amphotericin B; MFG, micafungin; 5-FC, 5-flucytosine; POS, posaconazole; VRC, voriconazole; ITC, itraconazole; and FLC, fluconazole.

### Adaptation to plants

As both *C*. *metrosideri* and *C*. *ohialehuae* were isolated from floral parts (stamens and styles, respectively) and most of their close phylogenetic relatives were originally found in plant material of tropical regions near the Pacific ocean (*e*.*g*. *C*. *ruelliae* [19], *C*. *heveicola* [49], *C*. *chantaburiensis* [47], *C*. *konsanensis* [48], and *C*. *vulturna* [20]), it can be hypothesized that these yeasts may be adapted to efficient growth on plant surfaces. In this regard, the phenotypic assays carried out in this study confirmed that both *C*. *metrosideri* and *C*. *ohialehuae* can assimilate the three main simple sugars present in floral nectar and other plant secretions (*i*.*e*. glucose, fructose, sucrose), and also other carbohydrates that are less abundant but fairly common in those habitats (*e*.*g*. xylose and maltose). Furthermore, both *C*. *metrosideri* and *C*. *ohialehuae* together with other closely-related species can grow in high osmotic (up to 60% w/w of glucose in most cases) and microaerophilic conditions, confirming that these yeasts can withstand some of the prevailing selective pressures of floral microhabitats [58–61]. Interestingly, none of these yeasts have ever been detected in nectar despite their capabilities of enduring nectar’s selective pressures, whereas other *Metschnikowiaceae* members such as *Metschnikowia reukaufii*, *M*. *gruessii* and *M*. *rancensis* have been identified as the main fungal inhabitants of floral nectar [62]. In this regard, it should be noticed that microhabitat heterogeneity can have an impact on the microbiota hosted by different flower organs [22]. Besides, the nectar of insect- and bird-pollinated plants often has different properties that select for specific microbial species [63]. As *M*. *polymorpha* flowers are visited by both birds and insects, it should be investigated if the relative frequency of visitation by each of these groups has any impact on its nectar yeast communities. Finally, in order to be dispersed from flower to flower, flower microbes need to be capable of surviving in/on the body of the animal visitors of flowers, which remains to be studied in most cases (but see Pozo et al., [64]). Therefore, the ability of *C*. *metrosideri* and *C*. *ohialehuae* to colonize and thrive in nectar, floral surfaces, other plant-associated habitats and the body of the animal visitors of flowers should be further assessed in future studies.

### Taxonomy

The description of the two new species presented in this paper is based on a single isolate, case of *Candida metrosideri*, or on multiple isolates retrieved from the same sample, case of *C*. *ohialehuae* (but note that, nevertheless, these *C*. *ohialehuae* isolates does not seem to be strictly clonal, as they presented D1/D2 intraspecific sequence variations and some phenotypic differences; see Tables 3 and 4 and S1 Table). Despite detailed inspection of the yeast isolates obtained from other flower samples, no additional isolates conspecific with *C*. *metrosideri* or *C*. *ohialehuae* were found. BLAST searches of D1/D2 LSU rRNA gene sequences obtained for the studied isolates demonstrated these diverged significantly from any other know species (6.5% for *C*. *metrosideri* and 9.7% for *C*. *ohialehuae*). Moreover, by analyzing the phylogenetic affiliation, it can be seen that both new species are close relatives of several emerging multi-drug resistant pathogens. Given the (phylo)genetic differentiation of *C*. *metrosideri* and *C*. *ohialehuae* with respect to other *Metschnikowiaceae* members and the decreased susceptibility of studied isolates to amphotericin B and some echinocandins, we consider that our taxonomic proposal is relevant enough.

#### Description of *Candida metrosideri* pro tempore Klaps, de Vega, Herrera, Junker, Lievens & Álvarez-Pérez 2020 sp. nov. [Mycobank accession: MB 832536]

Etymology: Specific epithet *metrosideri* [me.tro.si’de.ri. L. fem. s. *Metrosideros -i*], referring to the genus name of the host plant from which the yeast species was first isolated.

*Standard description*. This description is based on the characteristics of a single isolate (JK22^T^). On YM agar at 25°C after 48h colonies are white to cream, soft and butyrous, with an entire margin and a size ranging from 0.5 mm to 1 mm. Pseudohyphae or true mycelium are not formed. Cells are rounded to slightly ovoid (5–8 × 7–8 μm) and occur singly or in parent-bud pairs. The presence of vacuoles of different sizes is common, and cell budding is typically monopolar on a narrow base. Glucose and trehalose are weakly fermented. Galactose, sucrose, maltose, lactose, raffinose, melezitose, fructose, cellobiose, and melibiose are not fermented. Glucose, fructose, inulin, sucrose, trehalose, maltose, melezitose, methyl-α-D-glucoside (slowly), cellobiose, salicin, starch, L-sorbose (slowly), D-xylose (slowly), D-ribose, ethanol, glycerol, ribitol (slowly), xylitol, D-mannitol, D-glucitol, gluconolactone, 2-keto-D-gluconate, succinate, citrate (slowly), D-gluconate, D-glucosamine, N-acetyl-D-glucosamine, nitrate, nitrite (weakly), ethylamine, lysine, cadaverine, ammonium, L-ornithine, D-tryptophan, and urea are assimilated. Raffinose, melibiose, galactose, lactose, L-rhamnose, L-arabinose, D-arabinose, meso-erythritol, galactitol, myo-inositol, 5-keto-D-gluconate, DL-lactate, methanol, and hexadecane are not assimilated. Growth occurs in media containing 10% NaCl, 50% glucose, 0.01% cycloheximide, and 0.1% cycloheximide, but not 1% (v/v) acetic acid or 60% glucose. Growth is positive in vitamin-free medium (slowly), microaerobiosis, anaerobiosis (weakly), and at 10°C (slowly), 20°C, 25°C, 30°C, and negative at 4°C and 37°C. Tween 80 is hydrolyzed and arbutin is split. Acid production from glucose is weak. Gelatin and urea are not hydrolyzed, and no hemolytic activity is displayed.

The holotype JK22^T^ was isolated from the stamens of a *Metrosideros polymorpha* flower collected in the Hawai’i Volcanoes National Park (Hawaii Island, HI, USA), and is permanently preserved as metabolically inactive cultures, isotypes, in the CBS yeast collection of the Westerdijk Fungal Biodiversity Institute, Utrecht, the Netherlands (CBS 16091) and the Mycothèque de l’Université Catholique de Louvain (BCCM/MUCL), Louvain-la-Neuve, Belgium (MUCL 57821).

#### Description of *Candida ohialehuae* pro tempore Klaps, de Vega, Herrera, Junker, Lievens & Álvarez-Pérez 2020 sp. nov. [Mycobank accession: MB 832537]

Etymology: Specific epithet *ohialehuae* [o.hia.le.hua.’e. L. fem. s. *ʻōhiʻa lehua -e*], referring to the vernacular name of the host plant from which this yeast species was first isolated.

*Standard description*. This description is based on the characteristics of four isolates (JK58.2^T^, JK58.4, JK58.5, and JK58.6). On YM agar at 25°C after 48h colonies are white, soft and butyrous, flat or slightly domed, with a smooth or slightly wrinkled surface and entire margin. Colony size ranges from 1 mm to 2 mm. True mycelium is not formed. Cells are rounded to slightly ovoid (3–5 × 3–5 μm) and occur singly or in small groups. The presence of vacuoles is common, and cell budding is generally multipolar on a narrow base. Some isolates (JK58.2^T^, JK58.4, but not JK58.5 and JK58.6) form pseudohyphae after 8 weeks at 25°C on Custer’s chalk agar, but not on any other tested medium. Ascospores have not been observed in pure or mixed cultures on common sporulation media after eight weeks of incubation at 20 or 25°C. Glucose, fructose (latent), raffinose (weakly), sucrose (weakly), and trehalose (weakly) are fermented. Galactose, maltose, lactose, melezitose, cellobiose, and melibiose are not fermented. Glucose, fructose, inulin, sucrose, raffinose (slowly), galactose (slowly), trehalose, maltose, melezitose (weakly), starch, L-sorbose, D-xylose (slowly), glycerol, ribitol (slowly), galactitol (slowly), D-mannitol, D-glucitol, gluconolactone, 2-keto-D-gluconate, D-gluconate, D-glucosamine, N-acetyl-D-glucosamine, nitrate, nitrite (weakly), ethylamine, lysine, cadaverine, ammonium, L-ornithine, D-tryptophan, and urea are assimilated. Melibiose, lactose, methyl-α-D-glucoside, cellobiose, salicin, L-rhamnose, L-arabinose, D-arabinose, D-ribose, ethanol, meso-erythritol, xylitol, myo-inositol, 5-keto-D-gluconate, succinate, citrate, DL-lactate, methanol, hexadecane, are not assimilated. Growth occurs in media containing 10% NaCl, 50% glucose, 60% glucose (slowly), 0.01% cycloheximide, and 0.1% cycloheximide. All tested isolates split arbutin, produce acids from glucose, and can grow in microaerobiosis, anaerobiosis and vitamin-free medium (slowly), but not on media containing 1% (v/v) acetic acid. Growth is positive at 10°C (slowly), 20 °C, 25°C, 30°C, and negative at 4°C and 37°C. Gelatin, urea, and Tween 80 are not hydrolyzed, and no isolate shows hemolytic activity.

The holotype JK58.2^T^ was isolated from the styles of a *Metrosideros polymorpha* flower collected in the Hawai’i Volcanoes National Park (Hawaii Island, HI, USA), and is permanently preserved as metabolically inactive cultures, isotypes, in the CBS yeast collection of the Westerdijk Fungal Biodiversity Institute, Utrecht, the Netherlands (CBS 16092) and the Mycothèque de l’Université Catholique de Louvain (BCCM/MUCL), Louvain-la-Neuve, Belgium (MUCL 57822).

## Conclusion

The phylogenetic and phenotypic evidence provided in this study confirms that the studied isolates belong to the *Candida haemulonis* clade in the family *Metschnikowiaceae*, representing two undescribed species for which we propose the names *Candida metrosideri* pro tempore sp. nov. and *Candida ohialehuae* pro tempore sp. nov. The ecology and clinical relevance of these novel species should be further explored in future studies, as well as the causes and possible consequences of their decreased susceptibility to amphotericin B.

## Supporting information

S1 Fig Phylogenetic placement of *Candida metrosideri* and *Candida ohialehuae* based on DNA sequence analysis of the ITS1-5.8S-ITS2 rDNA region, as obtained by maximum likelihood analysis. Evolutionary distances were computed using the general time-reversible (GTR) model and are in the units of the number of base substitutions per site (see the scale). There was a total of 374 positions in the final alignment. The rate variation among sites was modeled with a gamma distribution (shape parameter = 0.18). Bootstrap node support values, based on 1000 replicates, ≥70% are shown next to the branches. Additionally, NJ (Kimura-2-parameter model, gamma distribution’s shape parameter = 0.18) bootstrap node support values ≥70% are marked by asterisks.
(TIFF)

S2 Fig Phylogenetic placement of *Candida metrosideri* and *Candida ohialehuae* based on DNA sequence analysis of the *RPB1* gene, as obtained by maximum likelihood analysis. Evolutionary distances were computed using the general time-reversible (GTR) model and are in the units of the number of base substitutions per site (see the scale). There was a total of 416 positions in the final alignment. The rate variation among sites was modeled with a gamma distribution (shape parameter = 0.27). Bootstrap node support values, based on 1000 replicates, ≥70% are shown next to the branches. Additionally, NJ (Kimura-2-parameter model, gamma distribution’s shape parameter = 0.69) bootstrap node support values ≥70% are marked by asterisks.
(TIFF)

S3 Fig Phylogenetic placement of *Candida metrosideri* and *Candida ohialehuae* based on DNA sequence analysis of the *RPB2* gene, as obtained by maximum likelihood analysis. Evolutionary distances were computed using the general time-reversible (GTR) model and are in the units of the number of base substitutions per site (see the scale). There was a total of 576 positions in the final alignment. The rate variation among sites was modeled with a gamma distribution (shape parameter = 1.26) and a proportion of invariant sites (0.51). Bootstrap node support values, based on 1000 replicates, ≥70% are shown next to the branches. Additionally, NJ (Kimura-2-parameter model, gamma distribution’s shape parameter = 1.26) bootstrap node support values ≥70% are marked by asterisks.
(TIFF)

S1 Table Complete phenotypic characteristics of *Candida metrosideri*, *Candida ohialehuae* and their close phylogenetic relatives. (DOCX)
