# Supplementary figures and images for: Candida metrosideri pro tempore sp. nov. and Candida ohialehuae pro tempore sp. nov., two antifungal-resistant yeasts associated with Metrosideros polymorpha flowers in Hawaii
Source: PLoS One. 2020 Oct 8;15(10):e0240093. doi: 10.1371/journal.pone.0240093 (PMC7544143; doi:10.1371/journal.pone.0240093)

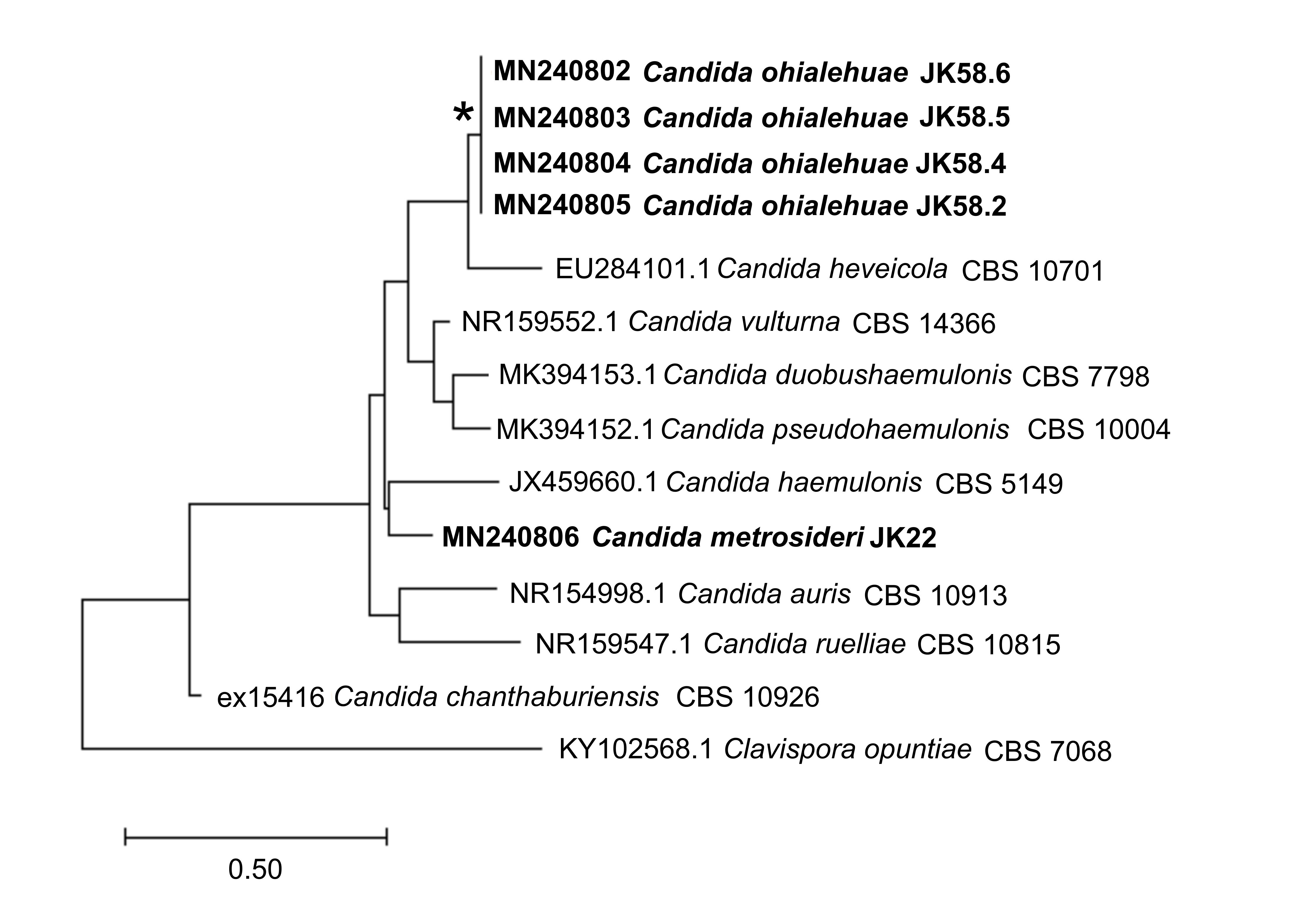

Supplement: S1 Fig — Evolutionary distances were computed using the general time-reversible (GTR) model and are in the units of the number of base substitutions per site (see the scale). There was a total of 374 positions in the final alignment. The rate variation among sites was modeled with a gamma distribution (shape parameter = 0.18). Bootstrap node support values, based on 1000 replicates, ≥70% are shown next to the branches. Additionally, NJ (Kimura-2-parameter model, gamma distribution’s shape parameter = 0.18) bootstrap node support values ≥70% are marked by asterisks. (TIFF) [file pone.0240093.s001.tiff]

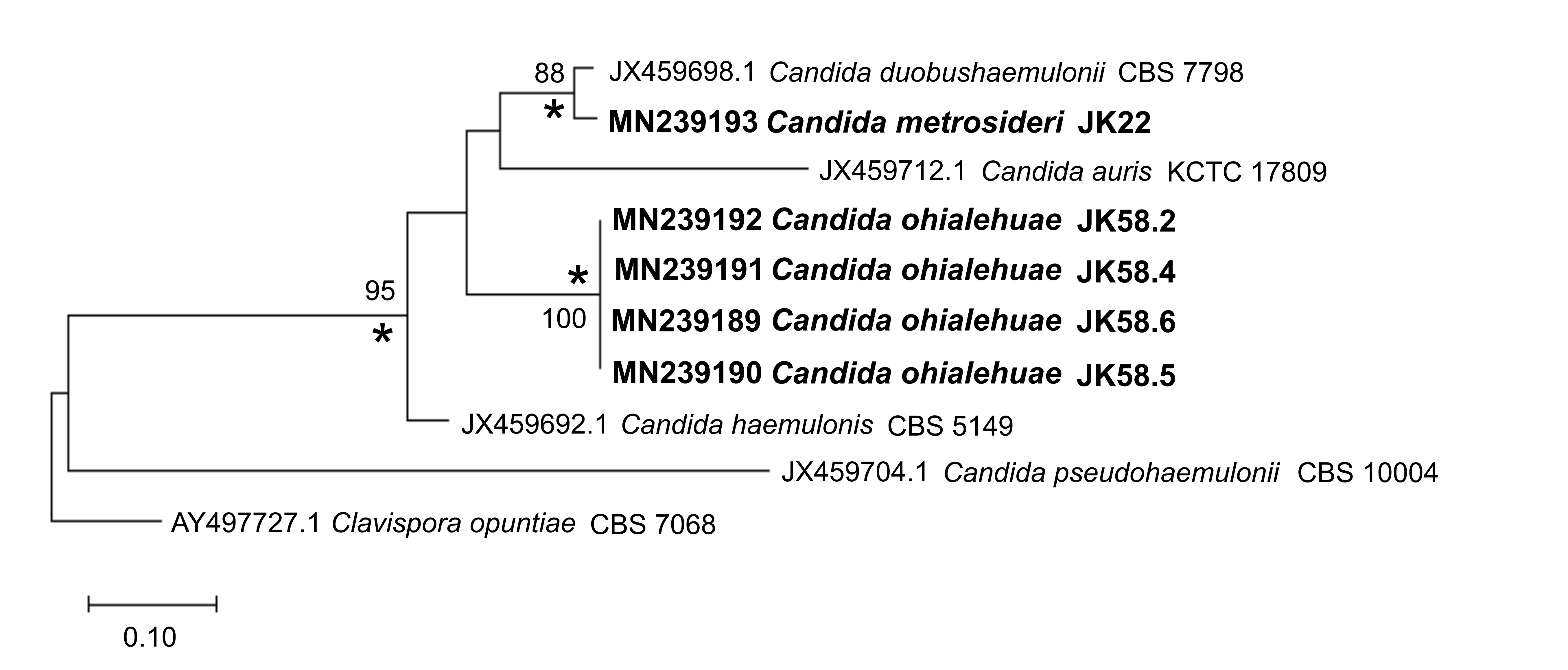

Supplement: S2 Fig — Evolutionary distances were computed using the general time-reversible (GTR) model and are in the units of the number of base substitutions per site (see the scale). There was a total of 416 positions in the final alignment. The rate variation among sites was modeled with a gamma distribution (shape parameter = 0.27). Bootstrap node support values, based on 1000 replicates, ≥70% are shown next to the branches. Additionally, NJ (Kimura-2-parameter model, gamma distribution’s shape parameter = 0.69) bootstrap node support values ≥70% are marked by asterisks. (TIFF) [file pone.0240093.s002.tiff]
