# Supplementary material for: Candida metrosideri pro tempore sp. nov. and Candida ohialehuae pro tempore sp. nov., two antifungal-resistant yeasts associated with Metrosideros polymorpha flowers in Hawaii
Source: PLoS One. 2020 Oct 8;15(10):e0240093. doi: 10.1371/journal.pone.0240093 (PMC7544143; doi:10.1371/journal.pone.0240093)
Supplement: S1 Table — (DOCX) [file pone.0240093.s004.docx]

**S1 Table. Complete phenotypic characteristics of *Candida metrosideri*, *Candida ohialehuae* and their close phylogenetic relatives.**

| **Characteristic** | **Reference strains** | | | | | | **7** | **8** |
| --- | --- | --- | --- | --- | --- | --- | --- | --- |
|  | **1** | **2** | **3** | **4** | **5** | **6** |  |  |
| **Fermentation** |  |  |  |  |  |  |  |  |
| Glucose | + | + | + | + | + | + | w | + |
| Galactose | - | ND | ND | - | - | - | - | - |
| Sucrose | + | + | ND | + | +/w | - | - | d/s |
| Maltose | - | ND | ND | d | - | - | - | - |
| Lactose | - | ND | ND | - | - | - | - | - |
| Raffinose | + | + | ND | - | - | - | - | w |
| Trehalose | + | ND | ND | ND | ND | - | w | d |
| Melezitose | - | ND | ND | ND | ND | ND | - | - |
| Fructose | ND | ND | ND | ND | ND | ND | - | d |
| Cellobiose | - | ND | ND | ND | ND | ND | - | - |
| Melibiose | - | ND | ND | ND | - | - | - | - |
| **Assimilation** |  |  |  |  |  |  |  |  |
| Fructose | ND | ND | ND | ND | ND | ND | + | + |
| Inulin | + | +/- | - | - | w | - | + | + |
| Raffinose | + | + | + | + | - | + | - | s |
| Melibiose | - | -/v | - | - | - | - | - | - |
| Galactose | + | + | + | + | + | + | - | s |
| Melezitose | + | + | + | + | + | + | + | s/+ |
| Methyl-α-D-glucoside | w/d/+ | -/w/d | v | w | + | w | s | - |
| Soluble starch | + | + | + | - | + | + | + | + |
| Cellobiose | -/+ | - | - | - | ND | ND | + | - |
| Salicin | -/w/d | - | - | + | - | + | + | - |
| L-Sorbose | - | + | d | + | + | + | s | + |
| L-Rhamnose | + | + | ND | - | + | + | - | - |
| D-Xylose | -/w/d | d | d | d | w | + | s | s |
| L-Arabinose | w/d/+ | -/w | v | - | d | + | - | - |
| D-Arabinose | -/w/d | d | - | - | w | - | - | - |
| D-Ribose | -/w/d | -/w | + | - | d | + | + | - |
| Methanol | - | w/d | - | - | - | - | - | - |
| Ethanol | - | w/d | d | + | d | - | + | - |
| meso-Erythritol | - | -/d | - | - | - | w | - | - |
| Ribitol | + | + | + | + | + | s | s | s |
| Xylitol | + | + | + | ND | - | ND | s | - |
| Galactitol | ND | ND | + | + | + | + | - | s |
| *myo*-Inositol | - | -/w | - | - | - | - | - | - |
| Gluconolactone | + | + | + | ND | + | + | + | + |
| 2-Keto-D-gluconate | ND | ND | + | ND | + | + | + | + |
| 5-Keto-D-gluconate | ND | ND | - | ND | - | - | - | - |
| DL-Lactate | - | -/w/d | ND | - | - | - | - | - |
| Succinate | + | + | + | + | + | + | + | - |
| Citrate | + | + | + | + | + | + | s | - |
| D-Gluconate | + | + | + | ND | + | + | + | + |
| D-Glucosamine | + | + | + | + | + | ND | + | + |
| *N*-Acetyl-D-glucosamine | ND | ND | + | ND | + | + | + | + |
| Hexadecane | ND | ND | + | - | - | ND | - | - |
| Nitrate | w | - | - | - | - | - | + | + |
| Nitrite | w | - | - | - | - | - | w | w |
| Ethylamine | - | + | + | + | + | + | + | + |
| Cadaverine | - | + | + | + | + | + | + | + |
| Ammonium | ND | ND | ND | + | + | ND | + | + |
| L-Ornithine | ND | ND | ND | ND | ND | ND | + | + |
| D-Tryptophan | + | ND | ND | ND | ND | ND | + | + |
| Urea | ND | ND | ND | ND | ND | ND | + | + |
| **Growth on YM agar at:** |  |  |  |  |  |  |  |  |
| 4°C | ND | ND | ND | ND | ND | ND | - | - |
| 10°C | ND | ND | ND | ND | ND | ND | s | s |
| 30°C | + | + | ND | ND | + | + | + | + |
| 37°C | + | + | + | + | + | w | - | - |
| 40°C | ND | - | - | ND | - | - | - | - |
| **Other phenotypic tests:** |  |  |  |  |  |  |  |  |
| Tolerance to NaCl (10% w/v) | ND | + | ND | ND | + | - | + | + |
| Osmotolerance (50% glucose, w/w) | + | + | ND | ND | + | + | + | + |
| Osmotolerance (60% glucose, w/w) | + | + | ND | ND | w | + | - | w/s |
| Gelatin hydrolysis | ND | ND | ND | ND | - | ND | - | - |
| Tween 80 hydrolysis | ND | ND | ND | ND | ND | ND | + | - |
| Acid production | + | - | ND | ND | - | ND | w | + |
| Tolerance to cycloheximide 0.01% (w/v) | - | + | ND | ND | + | - | + | + |
| Tolerance to cycloheximide 0.1% (w/v) | ND | ND | ND | ND | - | - | + | +/s |
| Tolerance to acetic acid (1% v/v) | - | - | ND | ND | ND | ND | - | - |
| Hemolysis | ND | ND | ND | ND | ND | ND | - | - |
| Growth in microaerobiosis | ND | ND | ND | ND | ND | ND | + | + |
| Growth in anaearobiosis | ND | ND | ND | ND | ND | ND | w | + |
| Splitting of arbutin | ND | ND | ND | ND | ND | ND | + | + |
| Hydrolysis of urea | ND | ND | - | - | - | - | - | - |
| Growth in vitamin-free medium | + | - | - | + | + | ND | s | s |

Strains: 1, *Candida vulturna* CBS 14366^T^; *Candida doubushaemulonii* CBS 7798^T^; 3, *Candida pseudohaemulonii* CBS 10004^T^; 4, *Candida heveicola* CBS 10702^T^; 5, *Candida konsanensis* CBS 12666^T^; 6, *Candida chantaburiensis* CBS 10926^T^; 7, *Candida metrosideri* JK22^T^; 8, *Candida ohialehuae* JK58.2^T^. Data for the reference strains originates from references [16,20,21,47,48] and the CBS database (<http://www.cbs.knaw.nl>). All isolates were positive for assimilation of D-glucose, sucrose, trehalose, maltose, glycerol, D-mannitol, D-glucitol, and L-lysine, and negative for assimilation of lactose. All isolates grow at a temperature of 25°C. Scoring system: +, positive; -, negative; d, delayed positive (latent); s, slowly positive; w, weakly positive; v, variable; ND, no data available.
